# Deep learning and multi-statistical features: an intra-frame forgery detection video method

**DOI:** 10.3389/frai.2026.1784359

**Published:** 2026-05-29

**Authors:** Diaa Uliyan, Manal Eid Alazmi, Mohammad Alsaffar, Moham’d Al-Dlalah, Meshari Alazmi

**Affiliations:** 1 Department of Information Security, College of Computer Science and Engineering, University of Ha'il, Ha'il, Saudi Arabia; 2 Department of Information and Computer Science, College of Computer Science and Engineering, University of Ha'il, Ha'il, Saudi Arabia; 3 High Diploma of Education, Arts and Humanities College, Applied Science Private University, Amman, Jordan; 4 Department of Artificial Intelligence, College of Computer Science and Engineering, University of Ha'il, Ha'il, Saudi Arabia

**Keywords:** deep learning, statistical features, VGG16, video forensics, video forgery

## Abstract

**Introduction:**

In this paper, we propose a technique for detecting spliced video forgeries using statistical clues extracted from the spatial and compression domains inside a suspicious video. The proposed technique employs a multi-feature architecture to train statistical features for every domain using the VGG-16 model. Examining the fused characteristics in specific image regions with great detail exposes instances of manipulation. Both the compression impacts of JPEG and the visual distortions that result from image modification are examined in our research.

**Techniques:**

In this study, we propose an alternative to the standard methods for detecting spliced forgeries in spatial domain textural analysis, such as entropy-based edges (MER), median filter residual (MFR), gray level regional maxima (FGM), morphological open images (MOI), and morphological erosion images (MEI). Derivative filters of image that may extract statistical information about manipulation traces from compressed data were chosen for artifact detection in double JPEG compression. As an example, the compression domain analysis makes good use of the 2D block Discrete Cosine Transform (DCT). There are three stages that comprise up the suggested method: (1) Video preprocessing splits videos into frames and converts each frame’s image to two domain formats: spatial and DCT compression. (2) Take each domain and extract several statistical characteristics. (3) Train VGG16 on a dataset to identify the spatially manipulated area in a video.

**Results:**

The proposed method has been validated and tested on the HTVD and GRIP datasets. The performance measure, such as Splicing forgery accuracy is 93.50% on the GRIP dataset and 92.40% in HTVD.

## 1 Introduction

The presence of affordable, high-quality video recording capabilities in mobile devices, along with numerous video-sharing platforms, plays a crucial role in the dissemination and exchange of visual content in everyday life. Visual information can serve as compelling evidence in a court of law to prove or support an individual’s statement during investigation. The authenticity of videos cannot be trusted due to the availability of advanced and intuitive video-editing tools. The advent of smart editing tools has facilitated information counterfeiting. Videos may be modified by including or eliminating objects/events, whether for benevolent or malevolent purposes (Munawar et al., 2023). Law enforcement agencies do not accept videos as evidence without forensic investigations. Not all instances of video tampering have the same impact; for example, altered media of a popular artist is less detrimental than manipulated video of a crime scene (Eltoukhy et al., 2025; Kumar et al., 2024). Video evidence is crucial for news reporting, intelligence agencies, insurance firms, copyright enforcement, and criminal investigations. Recent research concentrates on the forensic analysis of videos and images to verify the validity of visual content. This research is perpetual due to the continuous development of modern video editing technologies. The advancement of video editing profoundly influences our society. Despite the relatively few digital video forgeries, their occurrence have undermined public confidence in video content (Shelke and Kasana, 2021). The aim of video forgery detection is to ascertain authenticity and reveal probable alterations and forgeries, specifically to check if a video is legitimate or not and to perform localization (i.e., recognizing the forged region within the frame, neighboring frame (spatial tampering), or to ascertain exactly the frames have been added, substituted rearranged, or removed (temporal tampering) in the video). A variety of approaches have been suggested in (Javed et al., 2021; Ganguly et al., 2022; El-Shafai et al., 2024) for authenticating and identifying tampering in images; however, these methods are not directly applicable to videos for several reasons: (a) the majority of videos are encoded and compressed before storage and transmission due to the substantial amount of information in video frames; (b) The previously stated algorithms demonstrate considerable computing complexity when applied to video frames; furthermore, (c) temporal manipulation, such as frame insertion, deletion, replication, or transformation, remains undetectable by image fraud detection methods image fraud identification method. Various strategies have been proposed in the literature review for the identifying and localizing of video forgeries.

Illustrate that a video comprises a series of images, referred to as frames, which can be displayed in sequence to generate the illusion of motion, leveraging the stability of human sensory perception. A digital video is a three-dimensional sequence of images: two spatial dimensions ( x,y ) and one temporal dimension ( t ). The objective of video forensics is to determine clues of tampering to evaluate the validity and integrity of the video. Active techniques which have prior information (Swaraja et al., 2020) and passive (blind) techniques (Fadl et al., 2021) represent the two categories of video forensics approaches.

Ascertaining the legitimacy of surveillance video is a pressing issue. Intra-frame forgery is a widespread method for video editing. The forgery reduces correlation between adjacent regions at the tampering position. Thus, the correlation can be utilized to detect tampering. The algorithm’s key advantage is that it uses statistical feature extraction and anomalous pixel positioning. During the process of extraction, we obtain the 2-D statistical features of each frame because they are an important image attribute.

The next parts of this paper are structured as follows. Section 2 clarifies various types of video forgery attacks and provide specifics of the advanced video forensic detection methodologies. Sections 3 presented a forensic method to verify forgery in videos by exploiting its frames using spatial forgery (Intra-frame). In Sections 4 the datasets employed, result and comparative analyses are explained. Section 5 provides the conclusion outlines future directions.

## 2 Literature review

**Figure 1:**
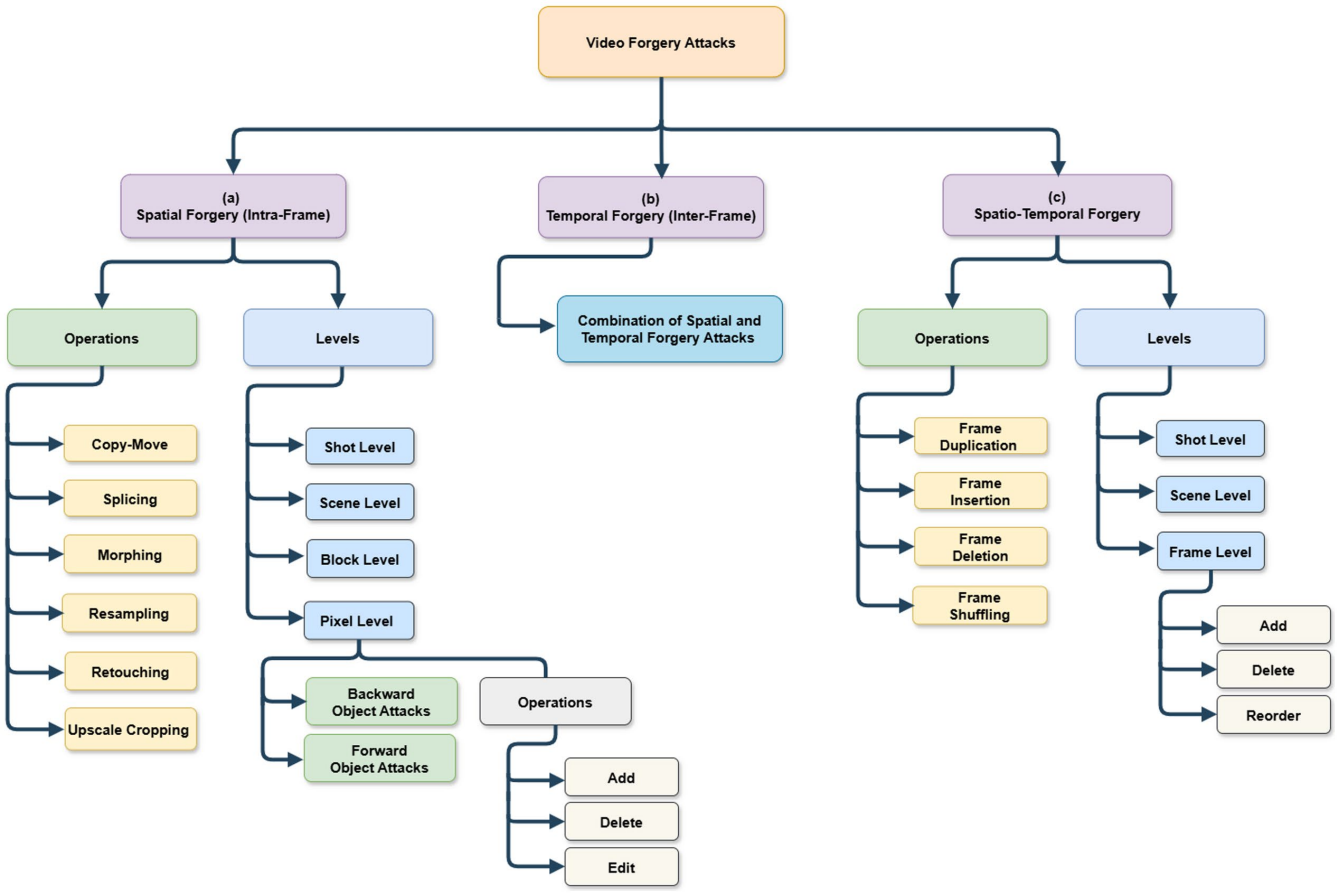
Video forgery approaches (reproduced with permission from Dhiman et al., 2023) **(a)** Spatial forgery operations called intra-frame (Patel and Sheth, 2021), **(b)** Temporal forgery operations called inter-frame (Yuan et al., 2021), and **(c)** Spatiotemporal forgery operations (Cedillo-Hernandez et al., 2018).

The primary aim of the literature is to conduct a comprehensive analysis and assessment of current digital video forensics technologies to understand recent advancements, potential future developments, and their implications for guiding the evolution of video forgery detection method (Sengar and Mukhopadhyay, 2017). Video forgery attack refers to the alteration of a video by the transformation or modification of its content. Three distinct categories of video forging methods are illustrated in Figure 1: (a) Spatial forgery operations called intra-frame (Patel and Sheth, 2021), (b) Temporal forgery operations called inter-frame (Yuan et al., 2021), and (c) Spatiotemporal forgery operations (Cedillo-Hernandez et al., 2018).

A substantial amount of video evidence is produced that can be scrutinized during a forensic analysis. This resulted in significant study in the domain of video forensics. The research studies conducted in the field of video forensics are the primary focus of this section. In recent reviews and surveys (Akhtar et al., 2022; Mohiuddin et al., 2023; El-Shafai et al., 2024; Vigneshwaran and Velammal, 2024), authors discussed the methodologies related to video forensics. This section, initially examined video forgery detection methodologies, followed by an exploration of information extraction approaches.

### 2.1 Spatial forgery (intra-frame)

**Figure 2:**
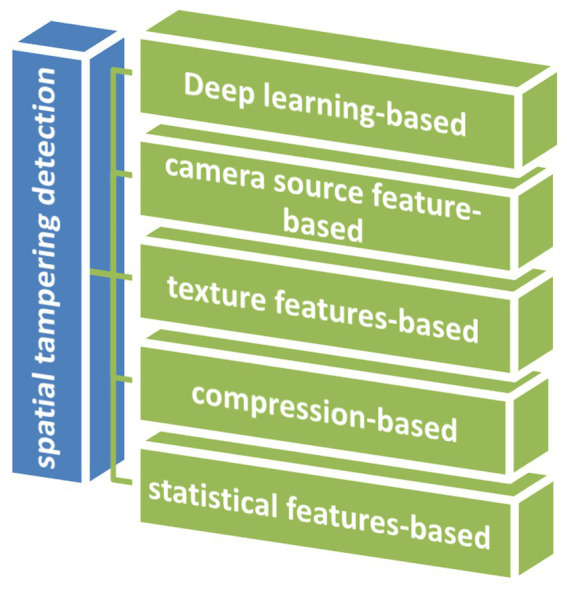
Video forgery approaches (Dhiman et al., 2023).

It utilizes the video sequence’s boundaries/regions in four levels (Short, Scene, Block, and Pixel Level). A forger can manipulate pixel in images per a video frame to attack the footage according to region, resulting in partial frame modifications. Tampering attacks involve inserting, deleting, cropping, and changing subject matter. This attack can be done with video editing tools (Fu et al., 2024). Intra-frame counterfeit processes include copy-move, splicing (Chen et al., 2022), retouching, re-sampling, morphing and upscale cropping (Vaishnav et al., 2024). Digital Forensic investigators have access to many sorts of information (artifacts or features) in videos to identify spatial tampering. These features categorize the approaches into the following classifications, as seen in Figure 2: (i) Deep learning-based approach, (ii) camera source feature-based approach, (iii) texture features-based approach, (iv) compression-based approach and (v) statistical features-based approach.

Zampoglou et al. (2019) employed Q4 and Cobalt filters in conjunction with trained beforehand ResNet and GoogLeNet models to facilitate the detection of spatial video counterfeiting. Two video datasets, designated as Dev1 and Dev2, are utilized to evaluate the methodology. The Dev1 dataset includes 30 authentic videos and 30 altered videos. In contrast, the Dev2 dataset contains 86 video pairs, comprising 44,000 frames and 134,000 frames, respectively. The combined results of Dev1 and Dev2 yield an accuracy of 85.09%, with a mean average precision of 93.69%.

A CNN was employed in (Yao et al., 2017) to acquire high-dimensional features, and the absolute difference between consecutive frames was utilized to mitigate temporal redundant information. A max pooling layer was integrated to reduce computational complexity, and a high-pass filter was applied to improve the residuals resulting from the tampering process. A total of 100 genuine videos and 100 fake videos are employed for the training and evaluation of the procedure. This method has achieved forged frame accuracy, pristine frame accuracy 89.90% and F1 scores of 94.07%.

A method for inter-frame forgery detection was developed in (Kaur and Jindal, 2020) utilizing a Deep Convolutional Neural Network. The methodology classifies video frames as either forged or legitimate through correlation analysis. The method was evaluated utilizing the REWIND and GRIP video datasets (Zhao and Zhang, 2020), achieving an accuracy of 98%. The approach exhibits significant accuracy; however, cross-validation is essential to validate generalization.

The proposed approach in (Kumar et al., 2022) utilizes a parallel CNN model to extract deep features. The system calculates the distance of the correlation coefficient derived from the deep features, facilitating the detection of separation between adjacent frames for the purpose of identifying video counterfeiting. The VIFFD (Nguyen and Hu, 2020) and SULFA (Qadir et al., 2012) standard datasets are utilized to assess the proposed methodology. The proposed method successfully differentiates between authentic videos and those that have been altered through insertion techniques. The achieved accuracy in frame-level forgery detection was 99.96%. In the domain of video-level forgery detection, achieved accuracies are 86.5% on the VIFFD dataset and 92% on the SULFA dataset.

### 2.2 Spatio-temporal forgery

Considering both spatial intraframe relationships and interframe temporal dependencies (Almestekawy et al., 2024). Kohli et al. (2020) developed a spatiotemporal video forgery identification method with CNN. Video frames are identified as either tampered or legitimate by temporal CNN, and then, the counterfeit within the video frames is pinpointed using spatial CNN. The model is trained using motion residual. The approach was assessed using the SYSU−OBJFORG dataset (Chen et al., 2015) and attained comparable results. Despite the method’s considerable efficacy, cross-data validation is necessary.

Fadl et al. (2021) suggested a method that employs spatiotemporal average (STP) on video frames and utilizes convolutional neural networks (CNN) to extract deep features, thereby minimizing the impact of post-processing operations (such as Gaussian noise, Gaussian blurring, brightness adjustments, and compression) through the use of pre-trained deep CNN models, specifically utilizing VGG-16 as the feature extraction tool, which yields superior results compared to prior hand-crafted features.

### 2.3 Temporal forgery (inter-frame)

The phrases inter-frame and intra-frame largely differentiate temporal tampering from spatial tampering. Inter-frame tampering operates at the frame sequence level where the pixels within particular frames remain unmodified, however the whole sequence is altered. A forgery can be performed by adding additional frames, deleting some of the frames from the video sequence, or altering the order of the frames based on the regional/boundary property of the video sequence (Su and Li, 2018; Dhiman et al., 2023). Intra-frame tampering occurs at the pixel level, when certain spatial regions are modified, although these adjustments are correlated to create a realistic counterfeit region.

Gowda and Pawar (2023) create a three-dimensional convolutional neural network (3DCNN) model to detect and localize video inter-frame counterfeiting using a multi-scale structural similarity index measurement technique. An absolute difference technique is used to distinguish video frames, efficiently minimizing temporal redundancy and detecting counterfeit artifacts within the frames.

Kingra et al. (2017) described a method for identifying inter-frame counterfeiting in camera surveillance. The suggested method detects frame insertion, removal, and replication in the MPEG-2 format and the H.264 video streams by leveraging prediction residuals and optical flow abnormalities. By integrating data from two forensic aspects, the approach can detect forgeries in a variety of films with different object movements.

In conclusion, there are some Difficulties in Identifying Forged Frame Boundaries where the Prior Work Gap is defined as follows: When a part of a video frame is edited or inserted (e.g., in a spliced video), it can be difficult to detect the exact boundaries of the forgery due to the lack of fine-grained feature analysis at the intra-frame level. The Current Solution: Advances in deep learning and feature extraction methods, such as convolutional neural networks (CNNs), are enabling more accurate identification of subtle discontinuities at the boundary of inserted content.

Intra-frame video analysis is needed in order to detect corruption occurring within a single frame, such as adding or removing objects or duplicating a portion of the frame. This is critical for determining the authenticity of videos used as evidence in litigation or for maintaining general media integrity, as such changes are simple to produce using standard software. In contrast to inter-frame forgery, which involves alterations between frames (such as deletion or duplication of full frames), intra-frame detection focuses on manipulations inside the visual content of each individual frame (Sengar and Mukhopadhyay, 2016a; Sengar and Mukhopadhyay, 2020).

## 3 Proposed method

**Figure 3:**
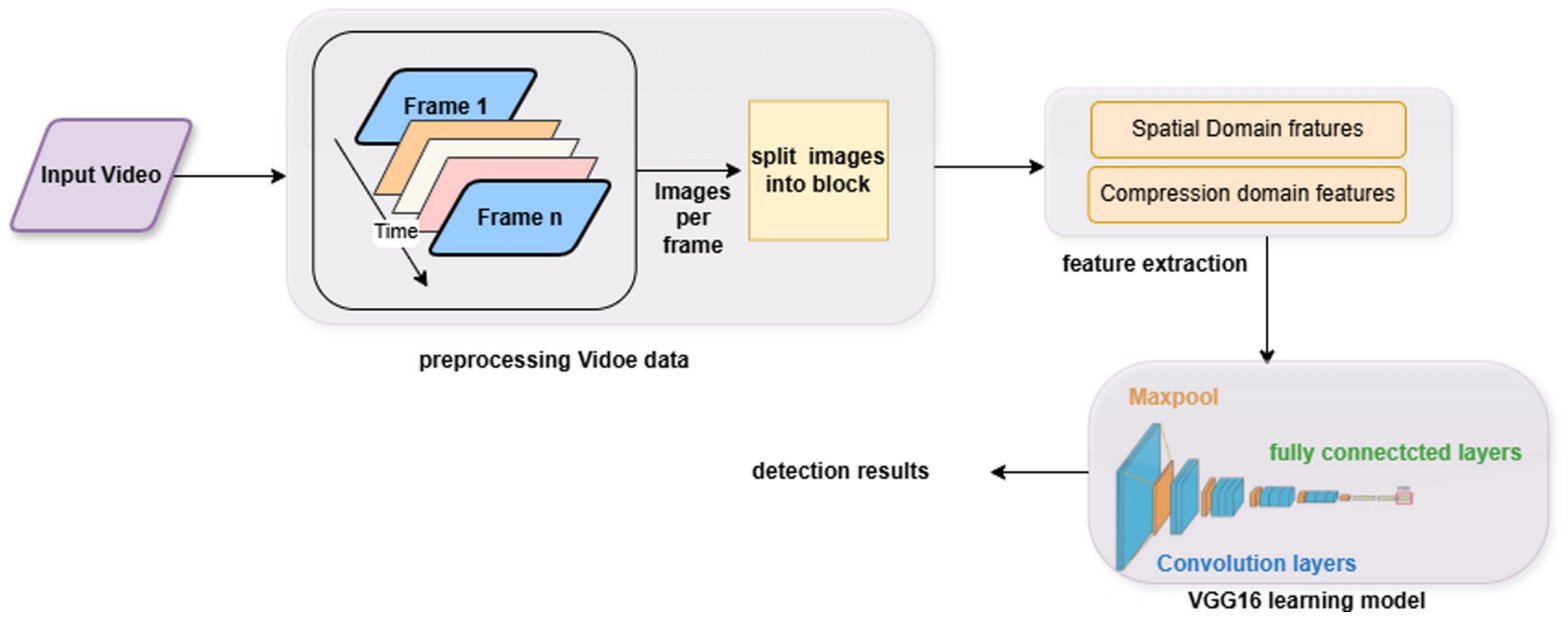
Spatial tampering detection approaches.

We suggest the video forgery detection model in this paper as a way to use statistical features from the spatial and compression domains in the images per frame itself. The suggested method trains multiple statistical features for each domain using a multi-features structure. It then reveals tampering by carefully looking at the fused features in regions. Our research looks at both the visual distortions arising from editing images and the compression effects that come from using JPEG. In this manner, a variety of forensic features can be examined during the training process. The type of manipulation method is splicing with different JPEG compression levels was used to make information that could be used for forgery analysis in reality. The paper presents a proposed method for detecting spliced forgeries by analyzing texture in spatial domain, beyond conventional techniques such as median filter residual (MFR), Find Gray level regional Maxima(FGM) , Entropy−based Edge(MER) , Morphological Open Image ( MOI ) and Morphological Erosion Image ( MEI ). To be able to detect artifacts from double JPEG compression, we chose to derive features that could extract statistical information about the manipulation traces from the compressed information. For example, the 2Dblock Discrete Cosine Transform(DCT) is used to be beneficial for the compression domain analysis. The main advantage of our method is detecting edges efficiently in visual contents by employing the concept of statistical entropy, which quantifies instability or disorder. Pixels at an edge have high local entropy because they represent a transition between separate, more structured regions, whereas pixels in a smooth region have low local entropy. Algorithms such as FGM, MER apply this notion to calculate local entropy values and identify edges where these values are high or change suddenly, making them more noise-resistant than other methods. Figure 3 gives Overview of employing multi-domain features for the video forensic model. The proposed approach comprises three steps as outlined below: (1) Video preprocessing divides videos into frames and transforms the images of each frame into two domain formats: spatial and DCT compression; (2) Extract multiple statistical features for each domain. (3) The VGG16 is trained on a dataset to classify the spatial forged region in a video.

### 3.1 Video preprocessing

Extracting particular frames from a video data is a vital task for many multimedia applications (Mohiuddin et al., 2023), including video analysis, editing, and processing. Videos primarily comprise a series of discrete images, referred to as frames, presented in quick succession to produce the perception of movement. The frame rate, or the number of frames displayed per second, is a critical determinant of video smoothness and quality. Standard frame rates comprise 24 frames per second (fps) for videos, 30 fps for standard TV, and 60 fps or greater for high-definition video. The pseudocode of extracting the frames of a certain video file using Python and OpenCV computer vision library (Bradski, 2000).

Images may be segmented into smaller regions to obtain appropriate information or statistical features on an individual basis (Sengar, 2019). The dimensions of these regions can be established according to defined specifications. Patches measuring 5×5, 8×8, and 16×16 are capable of delivering useful data. To ensure comprehensive coverage of the entire image, blocks or patches are typically overlapped, preventing any regions from being omitted. The process of dividing images into blocks utilizing the Python programming language and the OpenCV computer vision library.

### 3.2 Extracting statistical features

Splice manipulation of images is frequently implemented in video forensics. The texture investigation of the spliced regions is employed in this paper to identify video tampering. The local entropy and local range components are obtained from the suspected regions by utilizing the Median Filter Residual (MFR) in the spatial domain of the whole image. The ground truth mask’s (GTM) spatial domain feature set is extracted from the forged image’s local entropy using the MFR. This lets us include a rich information about the suspected regions: (a) Gray-level regional maxima (GRM) and (b) Entropy-based edges (EBE). The GTM extracts another spatial domain feature set from the local range component: (c) Morphological Open Image (MOI) and (d) Morphological Erosion Image (MOE). Subsequently, in the compression domain, we implement an M x M two-dimensional discrete cosine transform (DCT) (Dua et al., 2020; Sengar and Mukhopadhyay, 2016b) on the RGB pixels to examine local frequency features. It starts by transforming RGB pixels from suspicious regions into 2D block DCT features. Then it produces binarized coefficients utilizing the quantized DCT features of the Y channel. In real circumstances using JPEG compression, forgers compressed each manipulated image in a video to a quality factor ranging from 70 to 95.

#### 3.2.1 Spatial domain features

**Figure 4:**
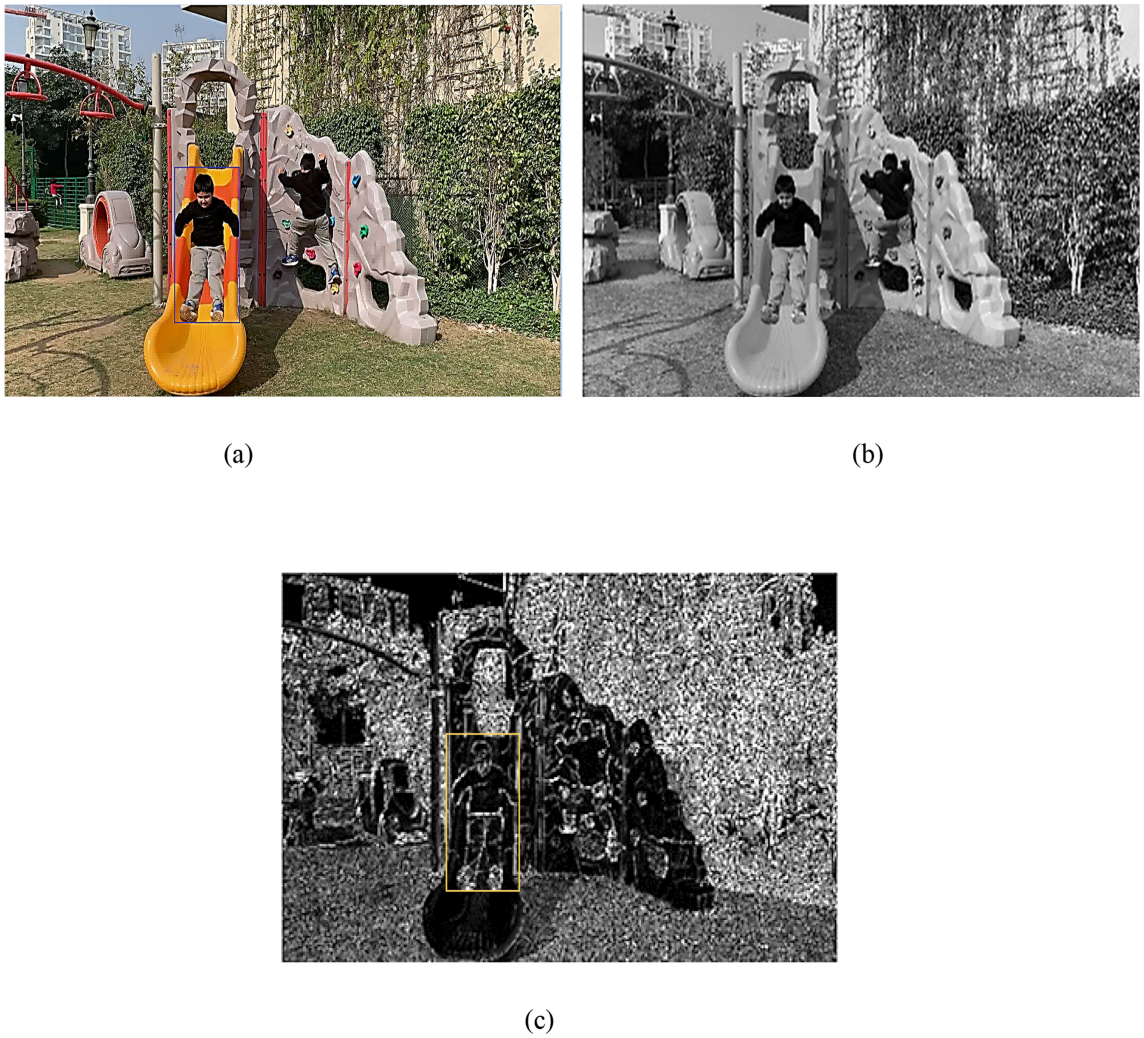
Median filtering residual as preprocessing step depicts the suspicion regions cut-paste region. **(a)** Spliced region surrounded by blue rectangle (in frame 39) of a tampered video (Singla et al., 2023) **(b)** Median filtered image **(c)** Autoregressive ( AR ) coefficients of MFR.

Median filtering is typically identified by MFR as a preprocessing step. Figure 4 (c) depicts the suspicion regions in image I by applying median filtering image (b), and then calculating the difference between image (a) and (b). Any portion of the image (a) is altered, it could show forgery clues in image (c). In Figure 4 (c), the MFR is expressed as Equation 1:

Difference(i,j)=∣I(i,j)−Median_filter(I(i,j))w∣ (1)

Median filtering is applied on Image I , where (i,j) represents spatial location and w signifies the window size of filtering. ∣.∣ represents the absolute function. The Autoregressive ( AR ) coefficients of MFR (Kang et al., 2012) in Figure 4 (c) is formalized as

$$M_{k}^{\left(\mathrm{row}\right)}=\mathrm{AR}(\text{mean}(\text{Difference}{\left(\;i,j\right)}^{\left(\mathrm{row}\right)})$$ (2)

$$M_{k}^{\left(\mathrm{col}\right)}=\mathrm{AR}(\text{mean}(\text{Difference}{\left(\;i,j\right)}^{\left(\mathrm{col}\right)}))$$ (3)

$$M_{k}=(M_{k}^{\left(\mathrm{row}\right)}+M_{k}^{\left(\mathrm{col}\right)})/2$$ (4)

The row and column directions are represented by row and col , respectively, whereas k is the AR order number, 1≤k≤p , and p is the maximum order number = 10 in our experimental results. The choice of p directly controls how much of the local correlation structure you capture. we need p in MFR for two cases:

1 Pixel correlations decay over distance: In natural images/videos, a pixel is strongly correlated with its immediate neighbors. This correlation decreases as you move further away where p determines how many neighboring pixels you consider to capture this correlation.
2 Feature richness vs. overfitting: Small p (e.g., 1–2): only captures very short-range dependencies. Might miss subtle tampering patterns. And Large p (e.g., 10+): captures long-range dependencies but risks including noise → AR coefficients become less stable.

Statistical measures such as entropy and range are employed in the classification of textures through texture analysis they can find edges that are more texture than brightness. The following equation is the basis for the calculation of entropy filter:

$$\text{Entropy}\;=-\sum_{l_{\min}}^{l_{\max}}\mu (l)\log_{2}\mu (l)$$ (5)

Where μ(l) is normalized by generating an intensity histogram H(l) for the image region and whose characteristics are to be precisely measured. If an intensity value is L(l=0,,,,1,,,,2,,,,…,,,,L−1) , the total pixel number is calculated by dividing the frequency of each intensity value by the total frequency (a pixel number of the image region) and then normalizing the result. A forger picks the region to be duplicated from the original image and transforms it into image. The Paste region’s transformation techniques include median filtering and JPEG compression. The forger attempts to circumvent the copyright issue with the tempered image, which wasn’t copied by masquerading as the original image. A tampered image using median and JPEG compression should be smooth, with minimal variance in gray-level data compared to the original. The suggested video forensics detection method should categorize the cut-paste region of a spliced image based on texture analysis. Each region demonstrates unique characteristics in terms of correlation, features, or validation among the surrounding pixels. A spliced image’s texture is studied by dividing MFR image into regions with different properties, such as the MFR image’s local entropy ( Le ) and local range ( Lr ). Le and Lr are computed as follows Equations 6, 7:

Le=Entropy(MFR), (6)

Lr=Range(MFR), (7)

Where Range is a range filter represents ( max−min ) value.

**Figure 5:**
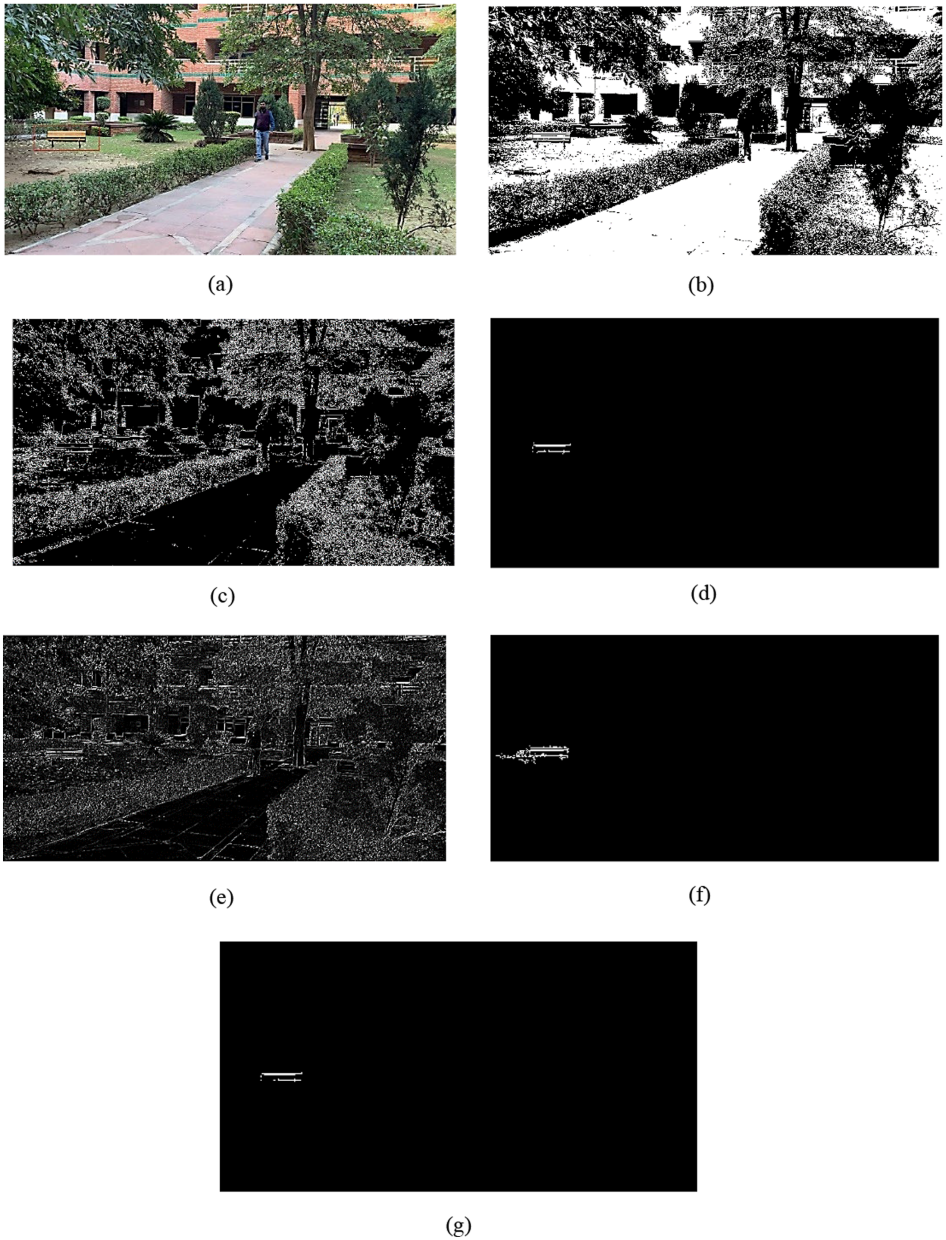
Spatial domain features of a forged video in HEVC-based tampered video dataset. **(a)** Forged cut paste region (in frame 50) of a tampered video (Singla et al., 2023). **(b)** Feature 1: Entropy image Le **(c)** feature 2: Range filter image Lr **(d)** feature 3: GRM image shows forged region in video frame; **(e)** feature 4: EBE image; **(f)** feature 5: MOIimage **(g)** feature 6: MEIimage.

A total of four ground truth mask features (GTM) presented in Figure 5 which label the Cut-Paste area in the spliced image. The GTM features are obtained through morphological opening mo and erosion me operation. The first GTM features is Gray Level Regional Maxima (GRM) of Closure through Image Restoration. it is denoted by Equation 8 as follows:

$$I_{e}=\text{Eros}(L_{r},r)$$ (8)

where $I_{e}$ represents the morphological erosion image.

$$I_{r}=\mathrm{Res}(I_{e},L_{r})$$ (9)

where $I_{r}\;$ is the restored image and r indicates the radius in pixel units for a morphological process. The GRM is defined by Equation 10:

$$\mathrm{GRM}=\mathrm{Reg}\_\max(\bar{\mathrm{Res}(\bar{\mathrm{Ie}},\bar{\mathrm{Ir}})})$$ (10)

Where Reg_max denotes the regional maxima function. The second texture features are then extracted using an entropy-based edge ( EBE ) method as

EBE=mo(I(i,j))·me(I(i,j))·Le(I(i,j)) (11)

Two types of ground truth masks are obtained using Equations 12, 13 and (13), respectively: morphological open images ( MOI ) and morphological erosion images ( MEI ).

MOI(r)=mo(Le,r) (12)

MEI(r)=me(Lr,r) (13)

##### 3.2.2 Compression domain features

**Figure 6:**
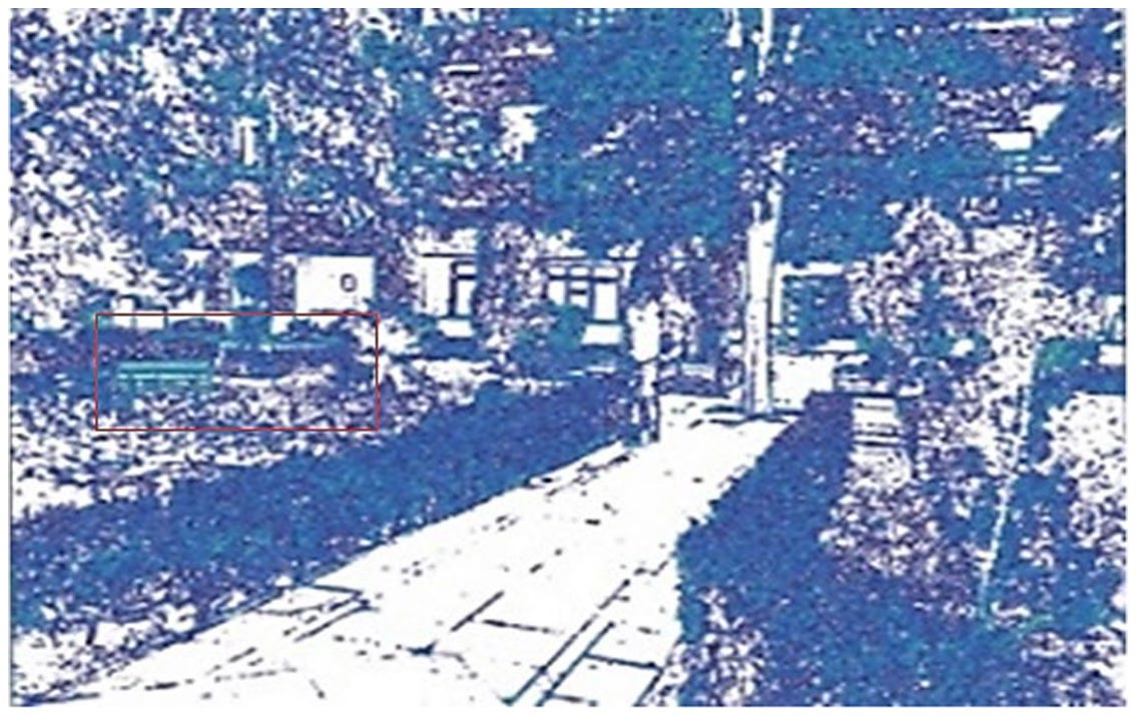
AC coefficients shows color changes in cut-paste region highlighted in red color.

for the fake image, those RGB are changed into new color sub images ( Y,Cb,orCr ). The sub-image with dimensions M x N is split into 8 × 8 blocks that are not overlapping, and a 2D−DCT operation (Dua et al., 2020) is carried out on each Block. There is only one DC coefficient and 63 AC coefficients in each block. The DC coefficient shows the average color of the 8×8 block as shown in Figure 6.

The 63 AC coefficients show how the color changes across the block. This method solely looks at the AC coefficients in order to investigate the color changes surrounding spliced regions in the tampered video. The AC coefficients are written in a zig-zag order and saved into feature vector as in Equation (14).

$$A_{i}={\left[a_{2,i},a_{3,i}\cdots a_{64,i}\right]}^{T},i\in \{1,2,\cdots N\}$$ (14)

Our main goal is to study the image’s behavior; hence each frequency must be analyzed independently throughout all N blocks. Then, we combine column vectors of N sub-image blocks to create a 63 × N matrix $M_{A}$ as follows

$$M_{A}=[A_{1}\;A_{2}\;\cdots \;A_{N}]={\left[\begin{array}{cccc} a_{2,1} & a_{2,2} & \cdots & a_{2,N}\\ a_{3,1} & a_{3,2} & \cdots & a_{3,N}\\ \vdots & \vdots & \cdots & \vdots \\ a_{64,1} & a_{64,2} & \cdots & a_{64,N} \end{array}\right]}_{63\times N}$$ (15)

Then, standard deviation $s_{k}\;$ is calculated and vectorized in a row wise manner as

$$S={\left[s_{2},s_{3}\cdots s_{64}\right]}^{T}$$ (16)

In this context, $s_{k}=1.5*\mathrm{MAD}(a_{k,i},a_{k,i+1}\cdots a_{k,N})$ is defined. It represents the standard deviation of the k^th^ AC coefficients. In a similar manner, the value of $o_{k}$ for each row is computed and stored in a feature vector as follows:

$$O={\left[o_{2},o_{3}\cdots o_{64}\right]}^{T}$$ (17)

Where, $o_{k}=\sum_{i=1}^{N}\;((a_{k,i},a_{k,i+1}\cdots a_{k,N})==1)$ . It is defined by using sign function to each row of the matrix. Sign function can be calculated as

$$\mathrm{sign}(a)=\{\begin{array}{ll} +1 & \;\text{if}\;a>0\\ \;0 & \;\text{if}\;a=0\\ -1 & \;\text{if}\;a<0 \end{array}$$ (18)

Sub-image is cropped by removing four rows and four columns from the top left edge. Following this, all previously described processes for the uncropped version (i.e., steps (14)–(18)) are repeated to compute the $s_{c}$ vector and the $o_{c}$ for each row of the AC Coefficients matrix of the cropped sub-image. The generation of the feature vector for the sub-image occurs as follows

$$F_{\mathrm{sub}-\text{image}}=[\begin{array}{llll} S^{T} & O^{T} & S_{C}^{T} & O_{C}^{T} \end{array}]$$ (19)

The final feature vector $F_{V}$ of the image is obtained by combining the feature vectors derived from the three sub-images (Y,,Cb,,Cr) in the following manner:

$$F_{V}=[\begin{array}{lll} F_{Y} & F_{\mathrm{Cb}} & F_{\mathrm{Cr}} \end{array}]$$ (20)

The suspected image under investigations might be authentic or fake. Let, $F_{V}^{A},F_{V}^{f}$ represents their corresponding feature vectors for authentic and fake images, respectively. Then difference vectors are then computed as

$$D_{V}^{\mathrm{AC}}=F_{V}^{A}-F_{V}^{f}$$ (21)

**Figure 7:**
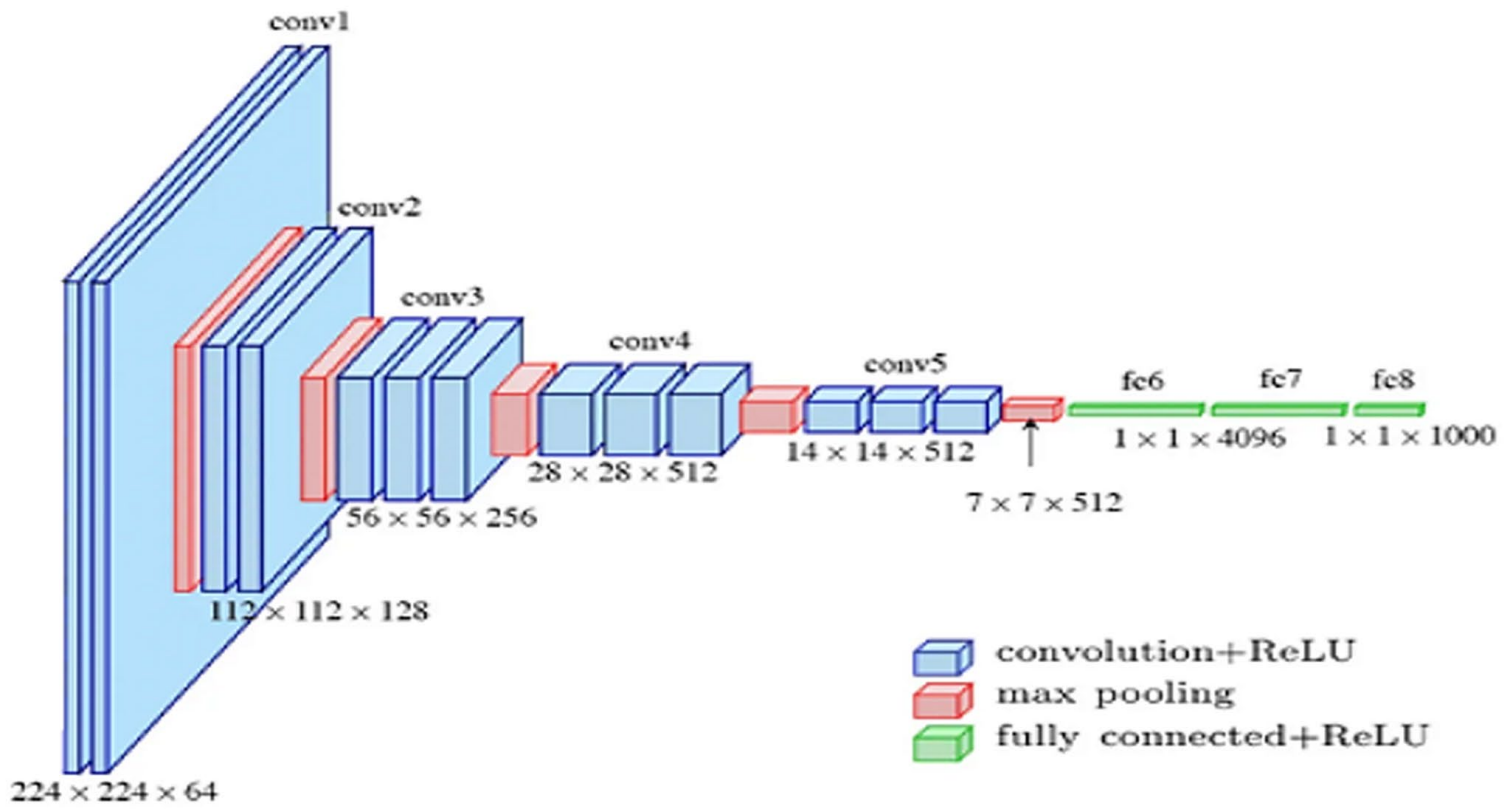
Architecture of VGG-16 learning model.

**Figure 8:**
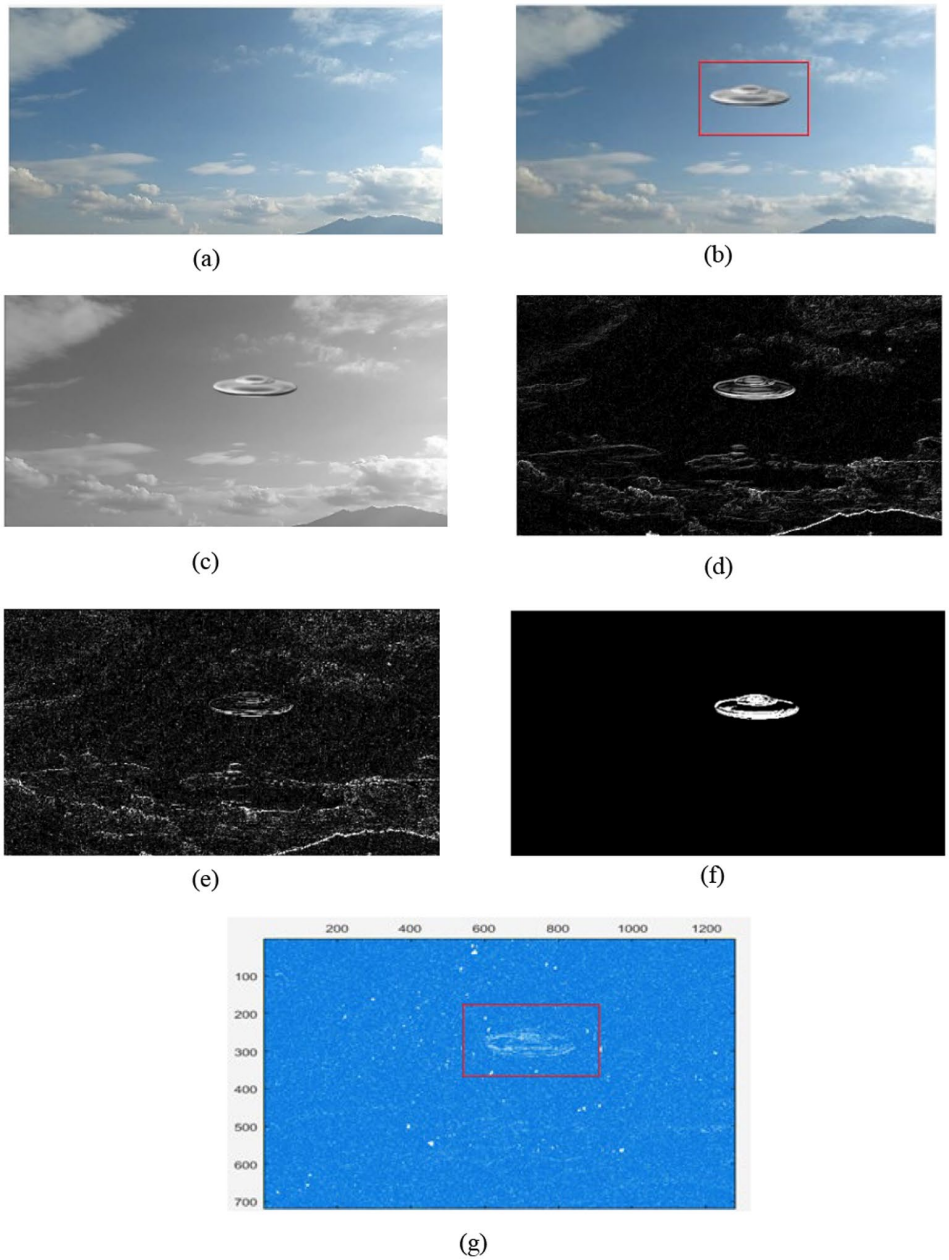
Experimental results on forged video “07_UFO”: **(a)** original video (frame 130), **(b)** spliced region (UFO) in forged video, **(c)** adaptive median filtering of forged region, **(d)** MFR of forged region, **(e)** Lr of forged region/frame in video, **(f)** GRM of forged region/frame in video, **(g)** 2D-DCT compression.

Thus, Intra-frame video analysis is indeed essential for detecting corruption within individual frames, especially in contexts like legal evidence or media integrity. Here’s the scenario of detecting forged regions in suspected videos defined as follows:

1. Get the suspected video and Identify alterations such as the addition, removal, or duplication of objects within a single frame using statistical features: (a) spatial domain and Compression domain feature. The features are extracted and saved as images as in Figures 7, 8 to ensure the authenticity of videos and it helps maintain trust in media by identifying manipulated content and identifies visual changes in the boundaries of objects to spot irregularities.
2. The dataset involves two classes of video: authentic videos and forged videos. The previous step cloud helps to categorize the extracted images from videos based on statistical features into: real or fake images. Then the original frames from videos are extracted into the authentic folder and forged frames are extracted into forged folder.
3. Splitting images for VGG16 typically involves dividing datasets into 80% training images/videos → used for training weights. 20% testing/validation sets to ensure authenticity of images in videos: Validation set: 10% → used to tune hyperparameters and monitor overfitting. Testing set: 10% → strictly for final evaluation.

### 3.3 VGG-16 model

Researchers at the University of Oxford’s Visual Geometry Group (VGG) and Google DeepMind collaborated to create the VGG network (Simonyan, 2014). The model placed second in the ILSVRC 2014 classification challenge and excelled in feature extraction. VGG model is a CNN model created for analyzing organized grid-like data, such as images. It consists of several layers, including convolutional, pooling, and fully connected ones. CNNs are extremely successful for applications such as image classification, object recognition, and segmenting images because of their multilayer feature extraction characteristics. It can be identified by its depth, which is composed of 16 layers, comprising 13 convolutional layers and 3 fully connected layers as in Figure 7. VGG-16 is well-known for its simplicity and performance, as well as its ability to perform well on a wide range of computer vision tasks. The employed model’s architecture as shown in Figure 7 consists of an accumulation of convolutional layers followed by max-pooling layers, with increasing depth.

The VGG-16 (Bagaskara and Suryanegara, 2021) enables intricate hierarchical representations of visual data, leading to enhanced and precise identification of forged areas. VGG-16, while simpler than more contemporary architectures, remains a widely utilized model in numerous deep learning applications due to its versatility and strong performance. The smaller convolution kernel demonstrates superior performance compared to the larger convolution kernel due to the multilayer nonlinear layer, which enhances network depth, facilitating the learning of more complex patterns while maintaining a lower parameter count. VGG-16, however, requires a greater amount of computational resources and parameters, resulting in higher memory utilization. VGG-16 comprises three fully connected layers, with the initial fully connected layer contributing the majority of the parameters. The images from the experimental dataset now eliminate the need for multiple feature extraction processes. As a result, the original VGG-16 will be combined with the full convolution model. The integration will result in a decrease in the model’s parameters and the quantity of layers in the fully connected layer. This method preserves the precision of feature extraction while simultaneously improving the training speed of the model. The model is based on the original VGG-16 architecture and incorporates the conventional full convolutional neural network framework. Initiate the architecture with a 32 × 32 convolutional neural network (CNN) layer, and subsequently incorporate a 2 × 2 max pooling layer after each convolutional layer. Following this, CNN layers are integrated with dimensions of 64 × 64, 128 × 128, 256 × 256, and 512 × 512, followed by a 2 × 2 max pooling layer. The convolution layer’s kernel size is decreased from 512 × 512 to 3 × 3, employing a stride of 2. This allows VGG-16 to process the input once instead of twice. Each convolution layer is followed by a nonlinear correction layer, resulting in the use of two nonlinear correction layers rather than one.

When more recent architectures like ResNet or EfficientNet are available, the use of VGG-16 in image-related tasks, such as video forensics, frequently raises concerns. Even though VGG-16 is regarded as “old” in comparison to these more recent architectures, there are still some strong arguments for its potential application. In certain situations, VGG-16 may still be chosen over ResNet or EfficientNet for the reasons listed below:

1 Ease of Use and Interpretability: With 16 layers, mostly made up of stacked convolutional layers with tiny 3×3 kernels and pooling layers, VGG-16 is a straightforward and uncomplicated architecture with a very regular structure. Compared to more complicated architectures like ResNet (which uses residual blocks) or EfficientNet (which uses complex design patterns and compound scaling), VGG-16 may be simpler to comprehend, interpret, and debug. In Forensics: VGG-16’s straightforward structure can be helpful in some forensics applications where interpretability is crucial (such as when forensics specialists wish to explain findings to a court). It is frequently simpler to determine where and why a model made a particular choice due to its simpler architecture.
2 Demonstrated Efficiency in Transfer Learning: The effectiveness of VGG-16 as a feature extractor in transfer learning tasks is well known. When pre-trained on massive datasets like ImageNet and optimized for particular tasks like video or image forensics, it performs admirably despite its relative simplicity. For many transfer learning tasks, it continues to be a dependable foundation because Large datasets have been used to pre-train it, and it has picked up practical low- and mid-level features like edges, textures, and basic shapes that can be helpful for a variety of vision tasks, including tampering detection. With comparatively little modification, it can be readily tailored to particular tasks (such as image or video forensics). The learned features in VGG-16 are frequently sufficient for many situations in forensics, where it is essential to identify even the smallest discrepancies, such as pixel-level anomalies.
3 Legacy and Usability: For many years, VGG-16 has been a mainstay of deep learning research. Due to its established reputation in the field, VGG-16 may have been used in numerous scholarly publications and earlier forensics-related work. Because of this legacy, it is simpler to duplicate or expand upon earlier research, particularly for scientists testing small adjustments or contrasting novel methods with a pre-existing standard. Starting with VGG-16 enables many researchers to preserve compatibility with earlier experiments, datasets, and work that was built around this architecture.
4 Small-Scale Dataset Performance: When the model is trained on smaller datasets, VGG-16 is comparatively less computationally demanding than deeper architectures like ResNet or EfficientNet. Because ResNet and EfficientNet typically need more data to achieve their potential benefits, they might not perform noticeably better in forensics tasks when the dataset is small. In Forensics: The simpler VGG-16 architecture may perform as well as, if not better than, more complex models in many video forensics tasks that involve specialized datasets that are relatively small in size (for instance, a dataset with only a few hundred tampered videos). On the other hand, in order to avoid overfitting, deeper models like ResNet or EfficientNet might need more data and processing power.
5 Reduced Parameters: VGG-16 has comparatively fewer parameters than more recent models such as ResNet or EfficientNet. In circumstances where computational efficiency is crucial or when achieving reasonable performance with less memory and computational overhead is the main objective, this can be advantageous even though it may be a drawback in terms of expressive power. In Forensics: The objective of real-time video forensics applications may be to swiftly analyze videos; the simplicity of VGG-16 enables quicker inference times. Additionally, it uses fewer resources, which could make deployment in some environments more practical.

## 4 Results

This section covers the experimental setup, performance evaluation, dataset used to test the proposed system, and results analysis. The approach is implemented on MATLAB 2020A and Python with a Core i7 6th Gen processor, 1 TB SSD, 16 GB DDR 4 RAM, and 6 GB GPU-NVIDIA GeForce GTX1050TI 4GB GDDR5 Graphics Card.

### 4.1 Experimental setup

The images extracted from the video were processed using a pre-trained deep learning model, specifically VGG-16. The VGG-16 architecture and specifications utilized in this study.

Table 1 summarizes the parameters utilized in the model. The Adam optimizer is employed to optimize the network’s parameters throughout the training process. The chosen learning rate is 0.001. A dropout ratio of 0.2 will be implemented to standardize the deep models. During the learning process, various batch sizes are evaluated within the range of [16, 32], with 32 determined to be the optimal batch size.

**Table 1:** VGG-16 experimental parameters.

Variable	Values
Learning rate	[0.001, 0.002]
Batch size	[16, 32]
Optimizer	[RMS_propagation, Adam, stochastic gradient descent]
Drop out	[0.2, 0.3]

### 4.2 Performance evaluation

The commonly used metrics necessary to evaluate the effectiveness of the proposed video forgery detection approach are defined as follows:

$$\mathrm{PR}=\frac{\mathrm{TP}}{\mathrm{TP}+\mathrm{FP}}$$ (22)

$$\mathrm{RR}=\frac{\mathrm{TP}}{\mathrm{TP}+\mathrm{FN}}$$ (23)

$$\mathrm{ACC}=\frac{\mathrm{TP}+\mathrm{TN}}{\mathrm{TP}+\mathrm{FN}+\mathrm{TN}+\mathrm{FP}}$$ (24)

$$F1\;\text{Score}=2\times \frac{\mathrm{RR}\times \mathrm{PR}}{\mathrm{RR}+\mathrm{PR}}$$ (25)

True Positive (TP), True Negative (TN), False Positive (FP), and False Negative (FN) are the terms used here. RR stands for recall, PR stands for precision and ACC for accuracy.

### 4.3 Datasets

#### 4.3.1 HEVC-based tampered video dataset (HTVD)

For more thorough testing capabilities, we use the HEVC-based Tampered Video Dataset (HTVD), which includes a variety of real and forged video scenarios. The dataset serves as a baseline for the researchers’ forensic investigative techniques, providing a diverse variety of realistic and carefully manipulated videos for validation and comparison. The HTVD dataset is made up of videos taken indoors, outdoors, and during surveillance. It contains 60 original videos, 966 altered videos, their ground truth information, and masks. All of the original videos were shot on smartphones that support HEVC (Singla et al., 2023). A diverse database of forged films is created by incorporating several forms of inter-frame forgeries such as frame insertion, frame deletion, and frame duplication, as well as object-based intra-frame forgeries such as cloning, splicing, and inpainting. Video forgery dataset is publicly accessible ^1^.

#### 4.3.2 GRIP dataset

In 2017, a huge standard dataset was introduced with 2520 altered images and 23 videos. The GRIP dataset of 10 faked videos was made by splicing them using Adobe After Effects CC. See (D'Avino et al., 2017) for a description of this dataset. This dataset is freely accessible online ^2^. The resolution of the videos is 720×1280 pixels. (D'Amiano et al., 2018) used Adobe After Effects Pro to perform copy-move forgeries in 15 videos, resulting in the GRIP dataset.

### 4.4 Experimental analysis

**Figure 9:**
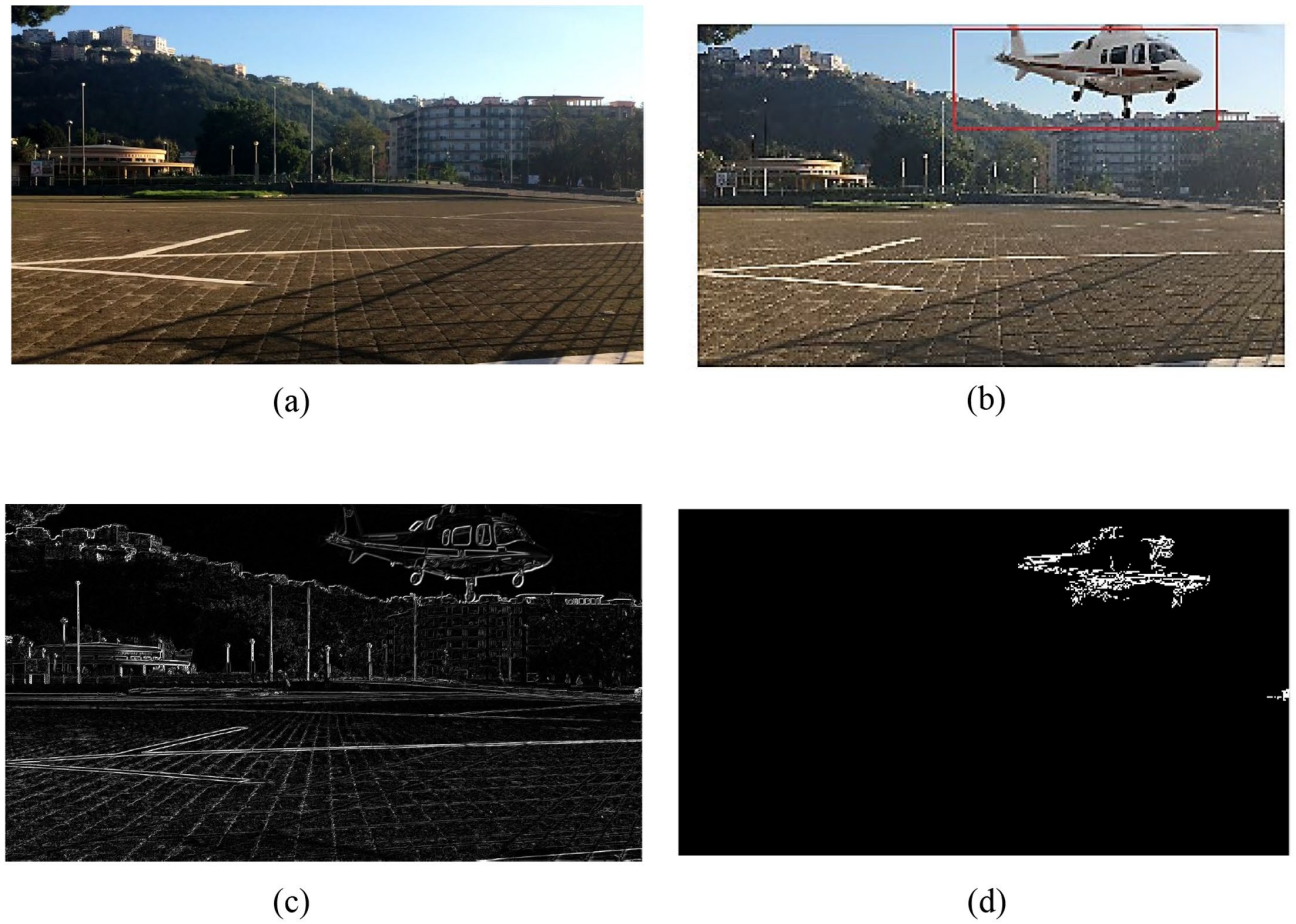
Experimental results on forged video “07_UFO”: **(a)** original frame from video (original image reproduced from D’Avino et al., 2017). **(b)** spliced frame (Helicopter) in forged video. **(c)** MFR of forged video. (d) GRM of forged region/frame in video.

Here, we showcase the experimental outcomes of the suggested approach on a set of videos that we import from HTVD and GRIP datasets, namely for videos (04_HELICOPTER and 07_UFO). Here are the findings of the experiment: Figures 8, 9.

Validation and testing of the suggested approach on the HTVD and GRIP datasets were accomplished. Table 2 displays the performance metrics, including ACC, PR, RR and F1-score. The splicing forgery accuracy is 92.40% in HTVD and on the GRIP dataset 93.50%.

**Table 2:** Evaluation of proposed method in two datasets.

Forgery type		Evaluation metrices (%)			
Splicing	Dataset	ACC	PR	RR	F1-score
	HTVD	92.40	92.80	92.90	92.88
	GRIP	93.50	93.90	93.70	93.80

VGG-16 model’s predictions can help identify GRM-highlighted regions that are actually genuine, i.e., not forged. Here’s a structured analysis:

1 VGG-16 typically trained for image classification, possibly fine-tuned for forgery detection. Outputs either class probabilities (forged vs. authentic) or feature embeddings. While GRM highlights regions in an image that appear anomalous, potentially indicating tampering. Often sensitive to image inconsistencies such as splicing, compression artifacts, or local noise discrepancies.
2 GRM may highlight regions that “look suspicious” visually but are actually genuine, because artifacts can arise from benign operations (compression, lighting changes, sensor noise). VGG-16, if properly trained, can act as a secondary process, potentially correcting false positives. Using VGG-16 to validate GRM regions by region-wise analysis:

1 Segment the GRM-highlighted regions
2 Convert the GRM map to a binary mask.
3 Each highlighted region becomes a candidate patch.
4 Feed patches to the VGG-16 model.
5 Crop or mask the original image using the GRM regions.
6 Pass each patch through the VGG-16 classifier.
7 Compare VGG-16 prediction with GRM suspicion

If a region is highlighted by GRM but VGG-16 predicts authentic with high confidence, it suggests a false positive in GRM. Conversely, regions highlighted by GRM and classified as forged by VGG-16 are likely true positives. Quantitative evaluation is performed. It helps quantify GRM’s precision improvement when combined with VGG-16.

To determine whether GRM-highlighted regions that appear suspicious are truly forged, by leveraging VGG-16 classification. Quantitative evaluation is performed as shown in Table 3. It helps quantify GRM’s precision improvement when combined with VGG-16.

**Table 3:** Quantitative evaluation of the proposed method

Metric (%)	With GRM only	With GRM + VGG −16
True positive rate (TPR)	88	93.50
False positive rate (FPR)	12	7
PR	89.20	93.40
RR	88.90	93.50
F1-score	89.22	93.45

It can be noticed that GRM often highlights areas that are visually inconsistent but not forged (e.g., JPEG artifacts, sensor noise). VGG-16 improves precision: Many false positives from GRM are filtered out when VGG-16 classifies the regions as authentic. Missed subtle forgeries: Very small or low-contrast forgeries may be highlighted by GRM but misclassified by VGG-16 if insufficient contextual cues exist. The interpretation of experiment: GRM alone is sensitive (high recall) but not precise. Adding VGG-16 reduces false positives, improving precision and F1-score, though it slightly reduces recall because some true regions may be misclassified.

The performance of the suggested system is evaluated on the HTVD and GRIP datasets using the approaches described in (Liu et al., 2018; Verdoliva, 2019; Li et al., 2023; Jin et al., 2022). Currently, the strategies can handle splicing forgeries one at a time. To ensure fairness, we tested different techniques on the same dataset and used same conditions. Table 4 shows that our technique has the most significant F1 and Accuracy compared to the other four methods. To ensure that the suggested method’s detection results remain unchanged, we employed a database of splicing regions with varying texture features.

**Table 4:** Test result of proposed vs existing methods.

Methods	Evaluation metrices (average %)			
	RR	PR	F1-score	ACC
Proposed method	93.30	93.35	93.34	92.95
Liu et al. (2018)	88.20	89.80	89.00	89.10
Verdoliva (2019)	86.40	88.00	87.20	87.30
Li et al. (2023)	90.00	92.50	91.20	91.40
Jin et al. (2022)	87.30	90.60	88.90	89.10

## 5 Conclusion

Detecting video forgeries remains a challenging task in multimedia security using passive techniques. The fabricated media has the capacity to sway the judge’s decisions in court on any legal issue. This research proposes a passive method for detecting forgeries (intra-frame forgery) in videos using the deep convolutional VGG-16 neural network and multi-statistical features. This approach can identify spatial forgeries such as splicing in an individual video. This approach has been tested on the HTVD and GRIP dataset.

Our approach exceeds existing techniques, detecting tampering in the video with an accuracy of 92.95% . Our future study will expand the method to detect more complex forgeries, such as deep forgery and frame duplication. Furthermore, our approach may be expanded to detect medical video forgeries when verifying medical video. The future work will focus on exploring more sophisticated fusion techniques, such as weighted fusion or attention-based mechanisms. It could potentially enhance the model’s ability to distinguish between authentic and forged regions by emphasizing more informative features.

## Data Availability

The datasets presented in this study can be found in online repositories. The names of the repository/repositories and accession number(s) can be found at: d.uliyan@uoh.edu.sa.

## Footnotes

1
https://drive.google.com/drive/folders/143NEyVjcHNVDDZVzIZm6nTEUk5-ezWb5?usp=sharing

2
https://www.grip.unina.it/download/prog/ForgedVideosDataset/Splicing/
